# Relationship between CD4 T cell turnover, cellular differentiation and HIV persistence during ART

**DOI:** 10.1371/journal.ppat.1009214

**Published:** 2021-01-19

**Authors:** Charline Bacchus-Souffan, Mark Fitch, Jori Symons, Mohamed Abdel-Mohsen, Daniel B. Reeves, Rebecca Hoh, Mars Stone, Joseph Hiatt, Peggy Kim, Abha Chopra, Haelee Ahn, Vanessa A. York, Daniel L. Cameron, Frederick M. Hecht, Jeffrey N. Martin, Steven A. Yukl, Simon Mallal, Paul U. Cameron, Steven G. Deeks, Joshua T. Schiffer, Sharon R. Lewin, Marc K. Hellerstein, Joseph M. McCune, Peter W. Hunt

**Affiliations:** 1 Division of Experimental Medicine, Department of Medicine, University of California, San Francisco, California, United States of America; 2 Department of Nutritional Sciences and Toxicology, University of California, Berkeley, California, United States of America; 3 The Peter Doherty Institute for Infection and Immunity, University of Melbourne and Royal Melbourne Hospital, Melbourne, Australia; 4 The Wistar Institute, Philadelphia, Pennsylvania, United States of America; 5 Vaccine and Infectious Diseases Division, Fred Hutchinson Cancer Research Center, Seattle, Washington, United States of America; 6 Division of HIV, Infectious Diseases and Global Medicine, Department of Medicine, Zuckerberg San Francisco General Hospital, University of California, San Francisco, California, United States of America; 7 Vitalant Research Institute and Department of Laboratory Medicine at the University of California, San Francisco, California, United States of America; 8 Medical Scientist Training Program & Biomedical Sciences Graduate Program, University of California, San Francisco, California, United States of America; 9 Infectious Diseases Section, Medical Service, San Francisco Veterans Affairs Medical Center, California, United States of America; 10 Institute for Immunology and Infectious Diseases, Murdoch University, Perth, Australia; 11 Center for Translational Immunology and Infectious Diseases, Vanderbilt University Medical Center, Nashville, Tennessee, United States of America; 12 Division of Bioinformatics, Walter & Eliza Hall Institute of Medical Research, Parkville, Australia; 13 Global Health Innovative Technology Solutions/HIV Frontiers, Bill & Melinda Gates Foundation, Seattle, Washington, United States of America; University of Pennsylvania, UNITED STATES

## Abstract

The precise role of CD4 T cell turnover in maintaining HIV persistence during antiretroviral therapy (ART) has not yet been well characterized. In resting CD4 T cell subpopulations from 24 HIV-infected ART-suppressed and 6 HIV-uninfected individuals, we directly measured cellular turnover by heavy water labeling, HIV reservoir size by integrated HIV-DNA (intDNA) and cell-associated HIV-RNA (caRNA), and HIV reservoir clonality by proviral integration site sequencing. Compared to HIV-negatives, ART-suppressed individuals had similar fractional replacement rates in all subpopulations, but lower absolute proliferation rates of all subpopulations other than effector memory (TEM) cells, and lower plasma IL-7 levels (p = 0.0004). Median CD4 T cell half-lives decreased with cell differentiation from naïve to TEM cells (3 years to 3 months, p<0.001). TEM had the fastest replacement rates, were most highly enriched for intDNA and caRNA, and contained the most clonal proviral expansion. Clonal proviruses detected in less mature subpopulations were more expanded in TEM, suggesting that they were maintained through cell differentiation. Earlier ART initiation was associated with lower levels of intDNA, caRNA and fractional replacement rates. In conclusion, circulating integrated HIV proviruses appear to be maintained both by slow turnover of immature CD4 subpopulations, and by clonal expansion as well as cell differentiation into effector cells with faster replacement rates.

## Author summary

HIV infection is a life-long disease for which we do not currently have a cure. One reason for this is that the virus goes into a dormant state and hides in CD4 T cell lymphocytes. Many of the less mature infected cells slowly self-renew (i.e., replace themselves) over time, whereas other types of infected cells rapidly proliferate and expand, and are replaced more rapidly. In our study, we directly measured how rapidly different types of CD4 T cells divide in treated people with HIV. In addition to confirming that less mature CD4 T cell populations self-renew at a very slow rate, and that more mature cells proliferate and expand at a more rapid rate, we also found that cell types that divide more rapidly are more likely to contain HIV. By carefully defining how rapidly these different cell populations are replaced, we help inform studies of HIV cure interventions that specifically target these discrete populations of HIV-infected cells.

**Summary**

HIV persistence is maintained by slow turnover of immature CD4 T cells and clonal expansion of more differentiated cells

## Introduction

The major obstacle to an HIV cure is the stable persistence of integrated proviruses within CD4 T cells during antiretroviral therapy (ART) [1–3]. While ART prevents active viral replication, reservoirs of replication-competent virus persist in latently-infected cells and viral rebound occurs when ART is discontinued [4].

The cellular composition of the HIV reservoir is thought to be influenced by homeostatic mechanisms governing the maintenance of a diverse CD4 T cell repertoire [5,6]. The size and distribution of the CD4 T cell population is dependent on the production of new progenitor cells, cell maturation, trafficking, and homeostatic as well as antigen-driven proliferation [5–7]. Naïve and stem-cell memory CD4 T cells, while less commonly infected than more mature subpopulations, may promote long-term viral persistence due to their renewal properties and longer survival [8–13]. More mature memory populations are enriched for HIV proviruses through undefined mechanisms [11,14]. Homeostatic cytokines (i.e., interleukin-7 or IL-7) promote thymopoiesis and the survival of progenitor cells (e.g., naïve and central memory cells) [15], while antigenic stimulation and other cytokines (e.g., IL-15) induce the expansion of more differentiated cells.

Given the central role of memory cell homeostasis in maintaining the reservoir, studies of T cell proliferation, differentiation, and death in treated HIV disease are needed. To date, most studies have focused on the turnover of bulk CD8 and CD4 T cell populations as measured by radioisotope labeling. Untreated chronic HIV infection has been associated with shorter half-lives amongst subpopulations of the bulk CD4 T cell population as well as lower production rates, which largely normalize after the initiation of ART [16,17]. Two distinct patterns of cellular accrual have been observed, with a biphasic pattern found in the memory/effector cell population, representing shorter-lived and longer-lived subpopulations, and a low-level proliferation rate in the longer-lived subpopulation, attributed to naïve CD4 T cells. The validation of new phenotypic markers that define various maturational stages of CD4 T cell subpopulations [18,19] now provides an opportunity to define the turnover of discrete subpopulations and to examine the relationship of such turnover to HIV persistence during ART.

Since HIV proviruses integrate into multiple unique positions in the cellular genome, integration site analysis can be used to identify cells that have clonally expanded from a single infected progenitor cell sharing the same integration site. Long-term ART-mediated viral suppression seems to select for genetically-identical HIV variants *in vivo* [20,21], and specific HIV integration sites (often in genes involved in cell proliferation and survival) have been associated with clonal expansion and persistence of infected cells in the periphery during effective ART, with up to 40% of distinct integrants being clonally-expanded and persisting longitudinally [22–24].

In this study, we used *in vivo* deuterium labelling via heavy water intake to define the turnover of resting CD4 T cell subpopulations in treated HIV infection. We also assessed how these parameters may affect HIV persistence as well as how they might be altered by the timing of initiation and the duration of ART.

## Results

### Participant characteristics

24 ART-suppressed HIV-infected individuals and 6 HIV-uninfected controls were recruited into this study. As we were not able to enroll enough female participants who initiated ART during very early infection, we restricted enrollment to men to avoid confounding by biological sex. While not significantly different, median age was slightly older in HIV-uninfected than in ART-suppressed participants (60 vs. 51 years, Table 1). As expected, HIV-infected participants had a lower median CD4 T cell count (573 vs. 647 cells/mm^3^ blood) and CD4/CD8 ratio (1.14 vs. 2.1) than uninfected individuals (p<0.034). Reflecting our sampling strategy, the ART-suppressed participants had a broad range of time to ART initiation (0.7 to 222 months from estimated date of infection) and duration of suppressive ART (1.2 to 18.8 years after initiation). HIV-infected participants had a median CD4 T cell count nadir of 335 cells/mm^3^ (2 to 664) and a median integrated HIV DNA level of 152 copies per million peripheral blood mononuclear cells (4 to 1,887).

**Table 1:** Participants characteristics.

Study Participants	Biological Sex	Age (years)	HIV infection status	Time to ART initiation (months)	Duration of ART (years)	CD4 Count (/mm3 blood)	CD4 Nadir (/mm3 blood)	CD4/CD8 Ratio	HIV RNA (copies/mL)	Integrated HIV DNA (copies/million PBMC)
**3693**	Male	46	Positive	0.9	1.2	841	468	0.94	< 40	4
**2641**	Male	60	Positive	4.2	2.8	341	252	0.74	< 40	53
**2609**	Male	33	Positive	1.7	1.5	899	518	1.51	< 40	129
**2647**	Male	33	Positive	4.5	3.4	532	486	1.34	< 40	294
**2531**	Male	51	Positive	1.9	3.4	1163	618	1.9	< 40	48
**2664**	Male	46	Positive	4.1	2.7	637	509	0.96	< 40	<63
**2606**	Male	29	Positive	1.7	3.5	787	341	1.68	< 40	563
**2454**	Male	35	Positive	0.7	7.1	513	352	1.31	< 40	25
**2661**	Male	54	Positive	3.4	12.9	739	442	1.02	< 40	463
**2522**	Male	42	Positive	4.4	6.4	341	203	0.81	< 40	485
**1473**	Male	55	Positive	34.9	4.5	573	311	1.32	< 40	1144
**1408**	Male	31	Positive	45.5	3.1	637	357	1.14	< 40	292
**1602**	Male	38	Positive	24.4	3.5	626	480	2.3	< 40	14
**3632**	Male	31	Positive	20.8	1.8	902	664	1.74	< 40	100
**1756**	Male	29	Positive	6.8	4.1	582	229	0.97	< 40	<76
**1597**	Male	56	Positive	187.7	2.6	432	335	0.6	< 40	247
**2610**	Male	53	Positive	34.8	2.6	468	393	1.07	< 40	1887
**2026**	Male	61	Positive	85.5	16.7	383	132	0.67	< 40	28
**2168**	Male	55	Positive	222	13.4	377	300	0.35	< 40	151
**2013**	Male	67	Positive	122.5	18.8	687	13	1.35	< 40	121
**2185**	Male	59	Positive	39.1	11.5	523	2	1.37	< 40	69
**2046**	Male	51	Positive	69.4	17	747	10	0.76	< 40	258
**2274**	Male	54	Positive	13.1	11.8	486	280	1.23	< 40	152
**2208**	Male	64	Positive	114.5	7.1	437	54	0.98	< 40	374
*Median*	*-*	*51*	*-*	*20*.*8*	*4*.*1*	*573*	*335*	*1*.*14*	*< 40*	*140*
*[IQR]*		*[33-56]*		*[4*.*1-69*.*4]*	*[2*.*8-11*.*8]*	*[437-739]*	*[203-480]*	*[0*.*81-1*.*37]*		*[56-354]*
**1806**	Male	58	Negative	NA	NA	736	NA	3.05	NA	NA
**1852**	Male	39	Negative	NA	NA	1570	NA	1.65	NA	NA
**1867**	Male	63	Negative	NA	NA	584	NA	1.42	NA	NA
**1943**	Male	61	Negative	NA	NA	1677	NA	2.28	NA	NA
**6532**	Male	62	Negative	NA	NA	957	NA	2.29	NA	NA
**6561**	Male	53	Negative	NA	NA	573	NA	1.99	NA	NA
*Median*	*-*	*59*.*5*	*-*	*-*	*-*	*847*	*-*	*2*.*1*	*-*	*-*
*[IQR]*		*[50-62]*				*[581-1597]*		*[1*.*6-2*.*5]*		

Abbreviations: ART, antiretroviral therapy; CD4, CD4+ T cell; CD8, CD8+ T cell; DNA, deoxyribonucleic acid; HIV, human immunodeficiency virus; IQR, 25–75% interquartile range; PBMC, peripheral blood mononuclear cells; RNA, ribonucleic acid.

### CD4 homeostatic proliferation is reduced in suppressed HIV infection

The proliferative capacity of resting CD4 T cell subpopulations was first assessed by comparing blood samples from 24 ART-suppressed HIV-infected (HIV+) to 6 HIV-uninfected (HIV-) participants. Enrichment of deuterium in saliva and plasma was measured repeatedly during the first month of the 45-day heavy water labeling period [25]. The fractional replacement rate of cells (fraction replaced per day) was estimated from *in vivo* deuterium enrichment in genomic DNA, corrected for time-averaged deuterium exposure in body water, from the following sorted resting (HLA-DR-) CD4 subpopulations: naïve (TN), stem-cell memory (TSCM), central memory (TCM), transitional memory (TTM), and effector memory (TEM) cells (see gating strategy in S1 Fig). Because CD4 T cell counts as well as CD4 T cell subpopulations proportions remained stable in periphery throughout the labeling period (S2A Fig), we can assume that the fraction of proliferating/dividing cells incorporating deuterium and entering the sampled pool is equivalently balanced by the fraction of cells leaving the pool. Label incorporation under steady-state conditions thereby allows calculation of cellular replacement rate and half-life within the sampled compartments (where half-life in the circulation is determined by any route for leaving the sampled pool, including cell death, differentiation into other phenotypes, or migration to different anatomical compartments).

**Fig 1:**
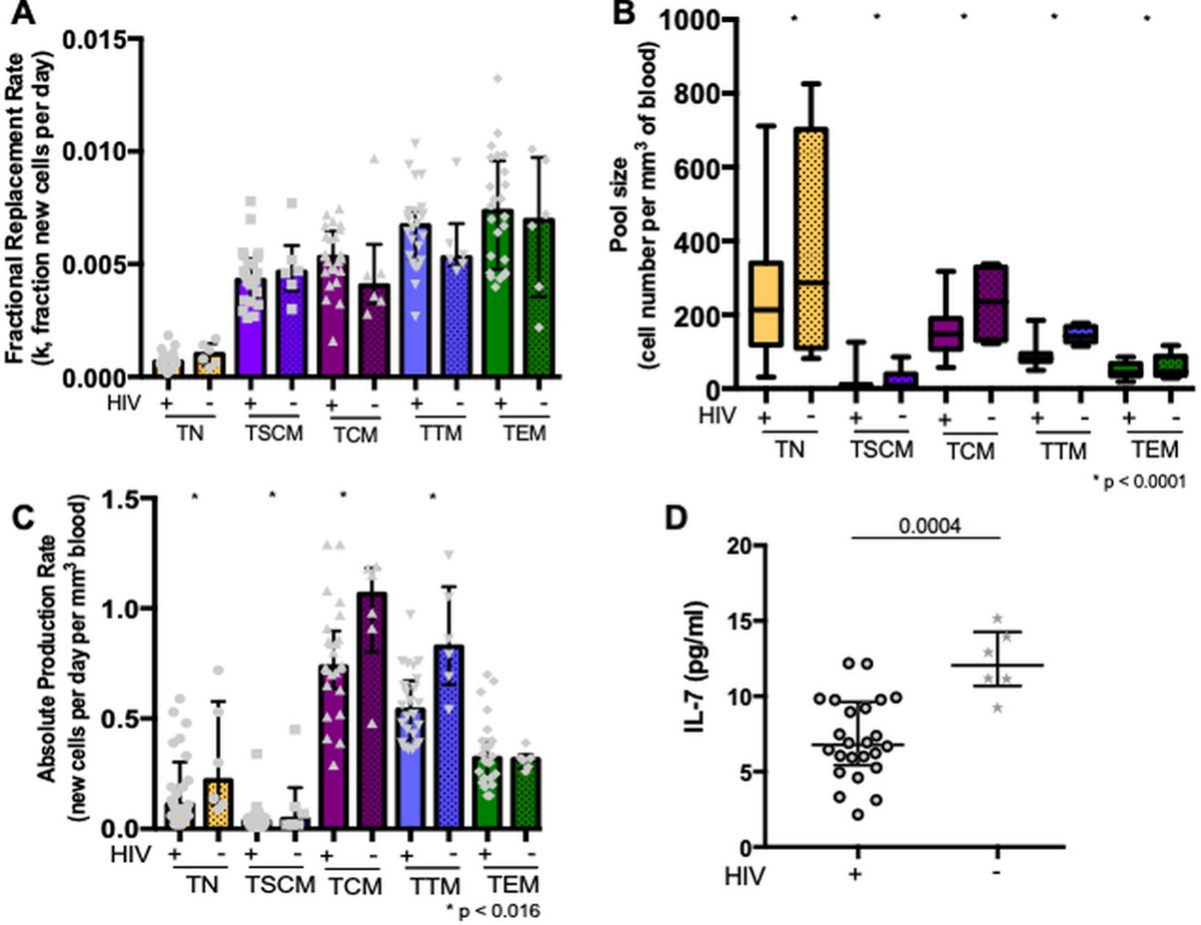
Absolute CD4 T cell proliferation and plasma IL-7 levels are decreased in treated HIV infection. Fractional cell replacement rates (fraction of new cells per day) and absolute proliferation rates (newly-divided cells per mm^3^ per day) were assessed in resting CD4 T cell subpopulations and compared between 24 HIV-infected (HIV+) and 6 uninfected (HIV-) participants in: naïve (TN), stem-cell memory (TSCM), central memory (TCM), transitional memory (TTM) and effector memory (TEM) cells. Medians and interquartile ranges are represented, and only significant *p* values are shown. **A.** The fractional replacement rate was inferred from the rate of incorporation of deuterium into the genome and is expressed as the fraction of the cells replaced per day (also called k, a constant rate with units day^-1^), and compared between HIV+ and HIV- participants across all subpopulations. **B.** The pool size was derived from measured CD4 T cell count and frequency in peripheral blood. The *p* value reflects the difference in pool size between HIV+ and HIV- across all subpopulations in a linear mixed model, which accounts for clustering between subpopulations within individuals. **C.** The absolute proliferation rate was calculated from the fractional replacement rate and pool size, as the number of cells produced per day per mm^3^ of blood. The *p* value assesses the difference between HIV+ and HIV- across TN, TSCM, TCM, and TTM subpopulations in a linear mixed model. **D.** Plasma levels of interleukin-7 (IL-7) (pg/ml) were compared between HIV+ and HIV- participants.

We focused our study on resting (HLA-DR-) CD4 T cell subpopulations since 100% of the HLA-DR+ CD4 T cell population turns over within 14 days (S2B Fig). Based on overall fractional synthesis (S2C Fig), the fractional replacement rates did not differ significantly between HIV- and ART-suppressed HIV+ participants for any resting CD4 T cell subpopulation (Fig 1A). The absolute cell counts were lower for all CD4 subpopulations in suppressed HIV+ compared to HIV- participants after controlling for age (p<0.0001, Fig 1B). There were no obvious differences in CD4 subpopulation frequencies in blood by HIV serostatus (S3A Fig). We then used the absolute cell counts, also known as pool sizes (cells per mm^3^ blood), and fractional cell replacement rates (fraction of new cells produced by cell division per day) to calculate the absolute proliferation rate of subpopulations (newly-divided cells per mm^3^ blood per day). Treated HIV+ were found to have a mean 45% lower absolute CD4 T cell proliferation rate across TN, TSCM, TCM, and TTM subpopulations compared to HIV- participants (p<0.016 from a linear mixed model assuming equivalent differences across subpopulations after adjustment for age as a continuous variable, Fig 1C), though rates appeared similar in the TEM population. Of note, absolute proliferation rates differed by CD4 T cell subpopulation among HIV-infected individuals.

### Homeostatic cytokine levels and thymic function

Since the impact of HIV infection on lowering the absolute proliferation rates was primarily observed within less mature CD4 T cell subpopulations that are maintained by IL-7 [26], we compared plasma IL-7 levels between groups. Compared to HIV- participants, ART-suppressed participants had significantly lower plasma IL-7 concentrations (6.8 vs. 12.1 pg/ml, p = 0.0004, Fig 1D). Nevertheless, there was no evidence for a correlation between plasma IL-7 levels and absolute proliferation rates or fractional replacement rates within any subpopulation among HIV+ participants.

As IL-7 may promote thymopoiesis as well as homeostatic proliferation in the periphery, we also assessed differences in thymic output between groups by measuring the levels of signal joint T-cell Receptor Excision Circles (sjTREC) in cell subpopulations. There was also no evidence of a difference in thymic output as reflected by relative or absolute sjTREC content in peripheral resting CD4 T cells between HIV+ and HIV- participants, even after adjustment for age (S3B Fig). Lastly, just as there were no differences in TEM absolute proliferation rates between HIV+ and HIV- participants, there were also no differences in plasma IL-15 levels, a cytokine that promotes effector cell proliferation and differentiation (S3C Fig).

### HIV is enriched in resting effector memory CD4 T cells with relatively short half-lives

**Fig 2:**
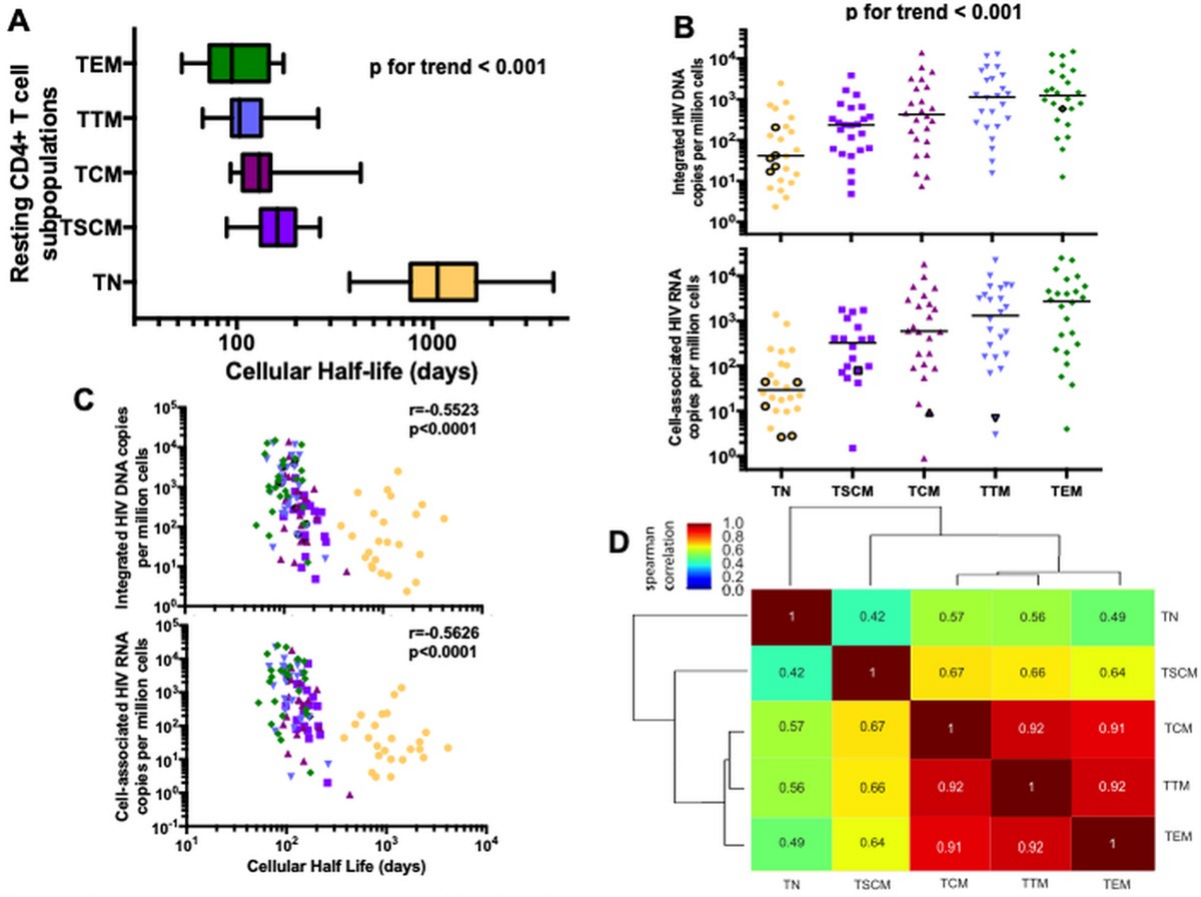
HIV is preferentially harbored in CD4 T cell subpopulations with relatively short half-lives. HIV genome levels and cellular half-lives were measured in 24 HIV-infected participants within the following resting CD4 T cell subpopulations: naïve (TN, yellow circle), stem-cell memory (TSCM, purple square), central memory (TCM, pink upward triangle), transitional memory (TTM, blue downward triangle) and effector memory (TEM, green diamond) cells. Medians and interquartile ranges are represented, and only significant *p* values are shown. Undetectable values were assigned a value equal to the threshold of detection based on the number of cells analyzed. **A.** The cellular half-life for each sorted cell subpopulation is shown in days. A *p* value for trend was statistically assessed across all five subpopulations. **B.** Levels of integrated HIV DNA and cell-associated HIV RNA were measured and are presented on a log10 axis as copy number per million cells. Medians are represented and each symbol represents a single participant sample. Values under the level of detection of the assay are represented in a black circle. A *p* value for trend was statistically assessed across all five subpopulations. **C.** The relationship between HIV genomes and cellular half-lives is shown for all cells. Integrated HIV DNA and cell-associated HIV RNA were measured and presented as copy number per million CD4 T cells. Cellular half-lives are expressed in days. **D.** Hierarchical clustering based on Spearman correlations among integrated HIV DNA frequency in each cell subpopulation.

From the measured fractional replacement rates, we calculated the cellular half-lives for each of the CD4 T cell subpopulations during ART-suppressed HIV infection as *t*_1/2_ = ln(2)/*k*, where *k* is the fractional replacement rate in day^-1^. In general, we found shorter half-lives with increasing cell differentiation (Fig 2A, p for trend <0.001). For instance, TN had the longest median half-life (2.9 years) while TEM had the shortest (3.1 months).

We next assessed the distribution of HIV DNA and RNA within cell subpopulations with defined half-lives. Integrated HIV DNA (intDNA) and total cell-associated HIV RNA (caRNA, using LTR primers) levels were measured within the same resting cell subpopulations (Fig 2B). Of all subpopulations, TEM were most highly enriched for both HIV intDNA (p<0.02) and caRNA (p<0.009) and, overall, infection frequencies increased with cellular differentiation (p for trend <0.001). Despite the greater abundance of naïve CD4 T cells in peripheral blood than other subpopulations, cells that were more differentiated also contributed a greater proportion to the total circulating intDNA (p<0.014) and caRNA levels (p<0.0003, S4A Fig) than those that were less differentiated. Across all subpopulations, shorter cellular half-lives were associated with higher total intDNA (r = -0.55 and p<0.0001) and caRNA levels (r = -0.56 and p<0.0001, Fig 2C), though this relationship was only significant within the TSCM subpopulation (S4B Fig). Interestingly, intDNA levels within CD4 T cell subpopulations tended to correlate with each other in a pattern consistent with linear T cell differentiation (Fig 2D). In an unbiased clustering analysis, intDNA levels within TN tended to correlate more strongly with those in TSCM than those in the more differentiated memory populations. Similarly, TCM, TTM and TEM intDNA levels tended to correlate more strongly with each other than with those in TN.

We also assessed the patterns of HIV transcriptional activity within each resting CD4 subpopulation using methods published previously [27], and assessed the following transcripts: U3-U5 “read-through” (suggesting transcriptional interference), TAR loop (transcription initiation), R-U5-tRNA-binding (“long”, 5’ elongation), U3-PolyA tail (“PolyA”, transcription completion), and multiply-spliced Tat-Rev (“Tat-Rev”, multiple splicing) (S5 Fig). While the pattern of transcriptional blocks was similar in the different cell subpopulations assessed, TN had lower levels of initiated (TAR) and 5’ elongated (long) HIV transcripts per provirus than each of the more differentiated TCM, TTM and TEM subpopulations (p<0.031). The levels of polyadenylated (PolyA) or multiply-spliced (Tat-Rev) HIV transcripts per provirus were similarly low in all CD4 subsets.

### Clonal expansion of HIV proviruses is associated with cellular differentiation

**Fig 3:**
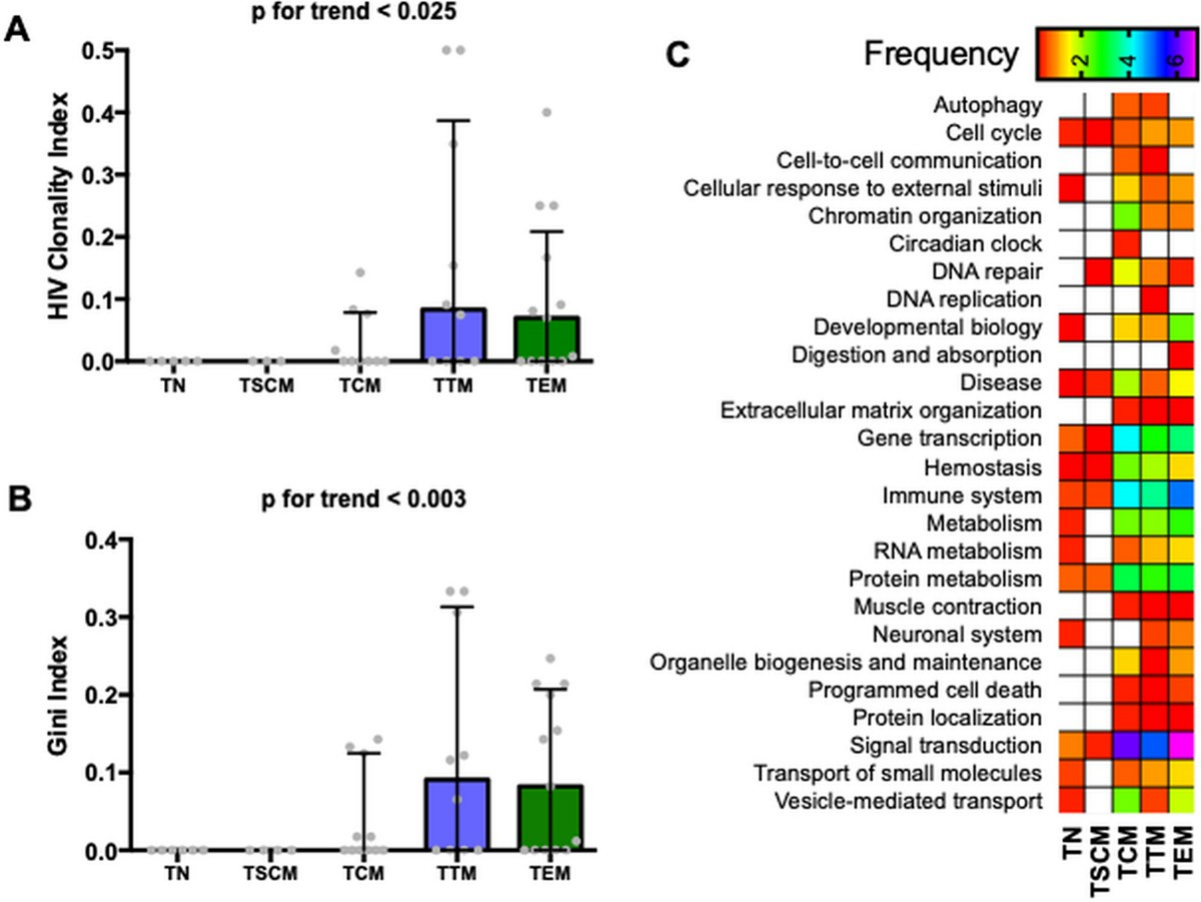
Cellular differentiation is associated with clonal expansion of HIV proviruses. HIV integration site analysis was performed on the following resting CD4 T cell subpopulations: naïve (TN), stem-cell memory (TSCM), central memory (TCM), transitional memory (TTM) and effector memory (TEM) cells. Medians and interquartile ranges are presented and only significant *p* values are shown. **A.** The HIV clonality index is calculated as the proportion of integration sites that are clonally expanded over the total number of integration sites detected. **B.** The Gini index is a measure of statistical dispersion intended to represent the integrated events distribution of an integration position, with values ranging from 0–1 from perfectly even (uniform clones) to highly skewed and uneven (many small clones and a few vastly expanded clones). **C.** Proportion of total unique HIV integration sites detected across all CD4 T cell subpopulations that fall within a given reactome (a set of linked genes, defined by an open source database of biologic pathways) and CD4 subpopulation. Specifically, the numbers reflect the total number of unique integration sites within a given reactome and CD4 subpopulation divided by the total number of integration sites detected across all CD4 subpopulations, multiplied by 100. Low frequencies are depicted in red while high frequencies are in purple.

To investigate the cellular mechanisms that contribute to the maintenance of HIV over time, we measured the clonality of HIV proviruses in each CD4 subpopulation. Integration sites were characterized in 12 of the 24 participants, and the total number of HIV integrants as well as the maximum clone size are presented in Table 2. In these events, cells deriving from clonal expansion were defined by a difference in amplicon length and at least three amplicons present. The HIV clonality index increased with cellular differentiation, from a median 0 in TN to 0.07 in TEM (p for trend <0.025, Fig 3A). We also calculated the Gini index, a test of statistical dispersion representing the degree to which clones are expanded in high numbers within each cell population. The Gini index tended to increase with greater cellular differentiation (p for trend <0.003, Fig 3B). Identical clones were sometimes observed in more than one CD4 subpopulation, often with greater degree of expansion in the more differentiated TEM subpopulation (S4C Fig). We also explored the types of genes, grouped by their known biological functions, in which unique HIV integration sites were detected. The biological function of the genes harboring HIV integrants were similar in multiple cell subpopulations, and were often related to cell biology, including immune processes and signal transduction (Fig 3C).

**Table 2:** Total number of HIV integrants and maximum clone size.

Participant ID	TN		TSCM		TCM		TTM		TEM		
	Number of integrants	Maximum clone size	Number of integrants	Maximum clone size	Number of integrants	Maximum clone size	Number of integrants	Maximum clone size	Number of integrants	Maximum clone size	Total integrants
1408					9	0	24	3	7	2	40
1473	8	0			4	0	25	0	43	5	80
1597	3	0			28	0	18	0	5	2	54
2046					14	3	28	0	2	0	44
2208	30	0	5	0	13	0	15	2	12	0	75
2454							3	2	253	3	256
2522	2	0	3	0	7	0					12
2531					2	0	3	2	11	3	16
2606									14	4	14
2610	53	0	6	0	57	3	86	2	47	3	249
2661							3	0	51	0	54
3632					152	3	140	6	8	0	300
Total	96		14		286		345		453		1194

### Early ART decreases cell fractional replacement rates and HIV reservoir size in resting CD4 T cells

**Fig 4:**
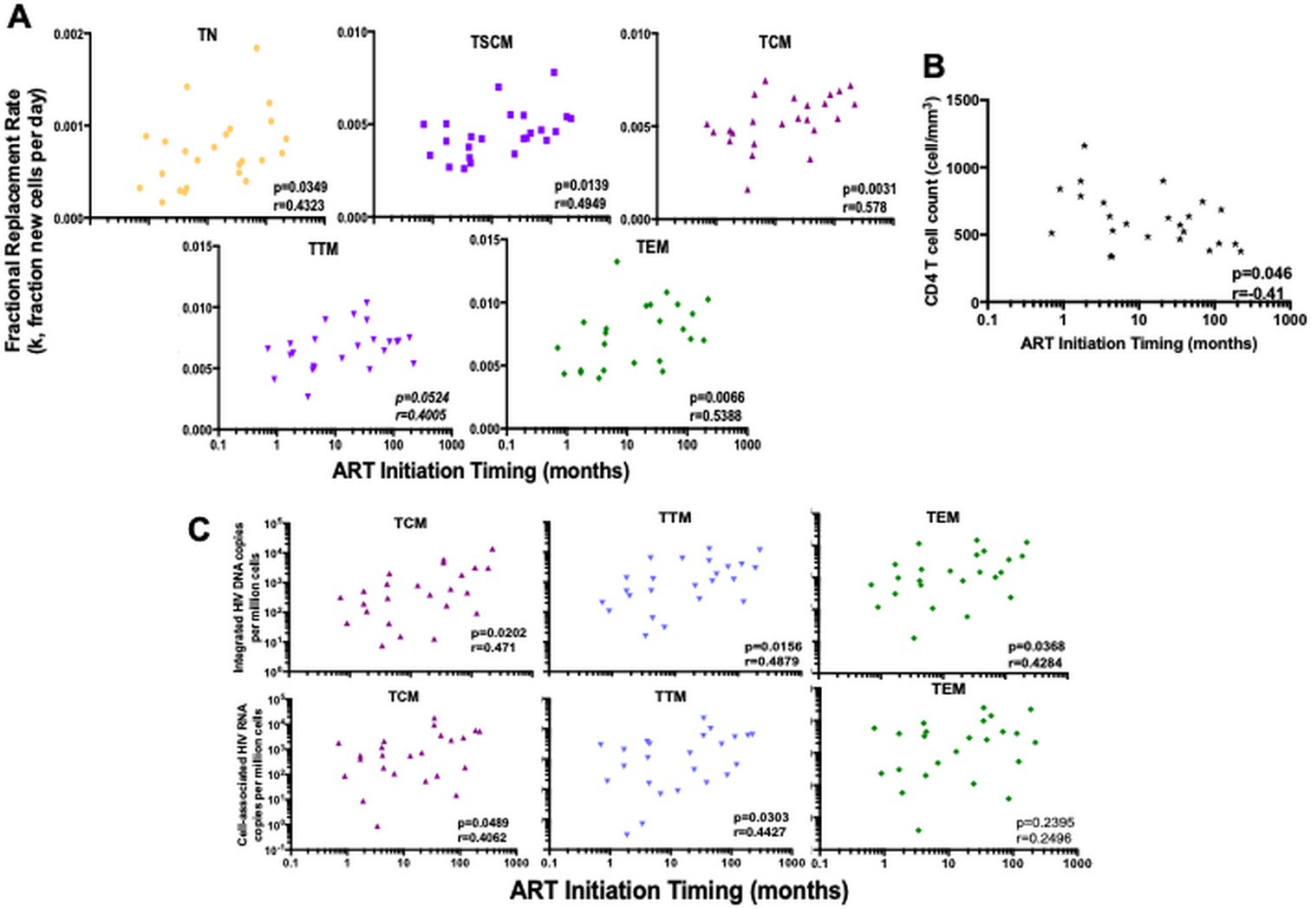
Early ART is associated with lower fractional replacement rate and HIV burden in resting CD4 T cell subpopulations. Measurements of the fractional cell replacement rate and HIV genome levels were performed in resting CD4 T cell subpopulations: naïve (TN), stem-cell memory (TSCM), central memory (TCM), transitional memory (TTM) and effector memory (TEM) cells. Each symbol represents a participant’s sample and the data are plotted on a log10-scale axis. Antiretroviral therapy (ART) initiation time is expressed in months. Spearman correlation coefficients (ρ) and associated p-values are reported in each subplot. **A.** The relationship between the fractional replacement rate and the initiation time of ART was assessed. The fractional replacement rate (k) is estimated as cells produced per day. **B.** The relationship between peripheral CD4 T cell count and the initiation time of ART was assessed. CD4 T cell count is estimated as cells per mm^3^ of blood. **C.** The associations between HIV nucleic acid levels and ART initiation time are shown for all participants. Integrated HIV DNA and cell-associated HIV RNA are measured and presented as copy number per million cells. Cell subpopulations that contribute most of the HIV reservoir are shown here, i.e., TCM, TTM and TEM. Undetectable values were assigned a value equal to the threshold of detection based on the number of cells analyzed.

We also assessed the impact of the timing of ART initiation on fractional replacement rates (inversely correlated to cellular half-lives) and HIV reservoir measures. Earlier ART initiation was associated with both lower fractional replacement rates (Fig 4A) and higher CD4 counts (Fig 4B), associations that remained significant after adjustment for age and ART duration. In cells that harbor the majority of proviruses (i.e. TCM, TTM, and TEM), later ART initiation was associated with higher levels of both integrated HIV DNA and cell-associated HIV RNA (Fig 4C), but not with IL-7 levels. Finally, we assessed the impact of the duration of ART on our outcomes, and found no evidence for a correlation between duration of ART and cellular fractional replacement rates (S6A Fig), HIV DNA, nor cell-associated HIV RNA (S6B Fig).

## Discussion

The persistence of HIV in resting CD4 T cells is the primary barrier to an HIV cure. While examples of clonal proliferation of HIV-infected CD4 T cells have been described [22,28–32], our study is the first to characterize the extent to which the proliferation and differentiation of resting CD4 T cell subpopulations contribute to HIV persistence. We made several important observations. First, the fractional replacement rates increased (while half-lives decreased) as cells matured from naïve to the effector memory phenotype, with rates similar between those with treated HIV disease and those who were uninfected. Second, ART-suppressed participants had lower absolute proliferation rates of less mature CD4 T cell subpopulations and lower IL-7 levels than HIV-negative individuals. Third, while naïve CD4 cells harbor less integrated HIV DNA than more mature subpopulations [12], they have extremely long half-lives and seem to be maintained by slow homeostatic proliferation in contrary to memory cells that have higher turnover. Fourth, we observed enrichment for clonal proviruses in more mature memory cells, suggesting that T cell differentiation and clonal expansion contribute meaningfully to the steady-state level of circulating HIV proviruses. Indeed, provirus levels were most strongly enriched in cells with the highest turnover rates. Lastly, earlier ART initiation was associated with lower fractional replacement rates of most CD4 T cell subpopulations, suggesting that the contribution of proliferation to HIV persistence is likely to be greatest in those who initiated ART at later disease stages.

Our finding that the fractional replacement rate of resting CD4 subpopulations is comparable in ART-suppressed HIV-infected and uninfected individuals mirrors findings in an earlier study [16]. By 12–36 months of ART, the fractional replacement rates of total CD4 and CD8 T cells tended to normalize, with greater production of cells rather than longer half-lives of circulating cells. In HIV-negative participants, *in vivo* labeling with deuterated glucose revealed that effector memory cells have higher turnover than central memory cells [33]. These results are consistent with ours using a deuterium labeling method that provides a much more reliable assessment of turnover in cellular populations like TN and TCM that turnover more slowly. Our observation of a substantially longer half-life in naïve cells than all other memory cells is in accordance with previously-reported data describing a biphasic accrual and decay kinetics in memory/effector but not in naïve cells [17]. Our study also provides novel data on the turnover of TSCM in the context of HIV infection. It was initially suggested, for instance, that stem-cell memory T cells were maintained through an enhanced capacity for self-renewal [34]. Stable isotope labeling in HIV-negative individuals demonstrated instead that they are maintained by ongoing proliferation, in accordance with our findings of a relatively shorter half-life close to that of memory cells [35]. Our study extends these observations to ART-suppressed HIV-infected participants.

Despite comparable fractional CD4 replacement rates, we found that ART-suppressed participants had lower absolute proliferation rates than HIV-negative participants in all CD4 T cell subpopulations except TEM, and also had lower plasma IL-7 concentrations. The earlier study discussed above also found lower absolute proliferation rates of CD4 T cells in treated HIV infection [16]. While untreated HIV infection has been associated with higher plasma IL-7 levels, in part due to increased production and decreased clearance in the setting of lymphopenia [36,37], the ART-suppressed participants with reconstituted CD4 compartments reported here had lower IL-7 concentrations than uninfected individuals. This could be due to a decreased production of IL-7 from lymphatic endothelial cells in secondary lymphoid tissues, presumably as a consequence of lymphoid fibrosis and collagen deposition following HIV infection [38,39]. Although a previous study showed that serum IL-7 levels normalized one year after the initiation of ART [40], we found lower levels in HIV+ participants here. While this difference could be due to the small sample size of the HIV-negative control group, it is worth noting that the cohort of participants recruited in this other study had low CD4 T cell counts while all of our participants had normalized CD4 T cell counts. It has been demonstrated that CD4 T cell lymphopenia and/or decreased CD127 (IL-7R alpha) availability drives IL-7 levels up as a consequence of increased production [41], decreased use, or CD127-mediated clearance [36].

Interestingly, the decreased absolute cell proliferation rates that we observed in treated HIV infection appeared to be limited to less mature CD4 subpopulations that generally express the IL-7 receptor specific alpha-chain, CD127, possibly suggesting a specific defect in homeostatic IL-7-driven proliferation despite preserved IL-15- and antigen-driven proliferation with differentiation. Additionally, those with earlier ART initiation had slower fractional replacement rates for most subpopulations than those initiating ART later, perhaps as a consequence of higher CD4 T cell counts, lower chronic immune inflammation and thus a reduced dependency on cell proliferation (whether homeostatic or antigen-driven). A practical inference is that the contribution of cell proliferation to HIV persistence seems to be greater among individuals who start ART at later disease stages.

Our assessment of HIV burden within naïve CD4 T cells is also consistent with findings of other reports confirming the persistent infection of naïve CD4 T cells, and in some studies, naïve T cell progenitors [11–13, 42–44]. Although harboring lower levels of total HIV DNA overall, naïve cells showed greater contribution of intact proviruses than central memory cells in chronically-infected participants [42]. While others did not find a consistent enrichment in CD4 T cells subpopulations [45], our observation of relatively long half-lives in naïve cells could be one important mechanism underlying the long term persistence of proviruses, as well as their potential to differentiate into other maturation stages upon stimulation. An earlier study demonstrated the production of as much virus from naïve cells as from central memory cells following in vitro stimulation with latency-reversing agents, further emphasizing the underestimated role of naïve CD4 T cells in HIV persistence [46].

Nevertheless, our integration site analysis only found expanded identical clonal integration sites in central, transitional and effector memory cell populations, suggesting that clonal proliferation and differentiation are more important mechanisms for viral persistence in memory than in naïve CD4 T cells. Consistent with different mechanisms of persistence in naïve and memory CD4 cell populations, we found much stronger correlations between memory CD4 T cell subsets in integrated HIV DNA and cell-associated HIV RNA levels than between memory and naïve CD4 cells. Collectively, these observations are consistent with a model whereby long-lived naïve cells are maintained by slow homeostatic proliferation resulting in a low HIV clonality index [47], while memory cells are supported by clonal expansion and antigenic proliferation resulting in larger clonally expanded HIV populations [48]. We recognize that other biological mechanisms might play a role including shared clearance mechanisms or cell differentiation. In previous studies, cellular proliferation has been implicated as a direct contributor to the clonal expansion of HIV proviruses in memory T cells, based on the expansion of identical HIV sequences and/or integration sites, largely in effector cell populations [22,29,49–57].

While infected naïve CD4 T cells are less likely than memory cells to undergo antigen-induced clonal proliferation, it is still plausible that at some point, they could also give rise to expanded memory cell progeny and contribute to the persistence of HIV genomes through both homeostatic proliferation and differentiation, even though we have not observed it here given our limited sampling. We also observed that resting HIV-infected naïve CD4 T cells were less likely to initiate HIV transcription than memory cells. Presumably, lower transcriptional activity might render infected naïve cells less susceptible to immune-mediated clearance and less responsive to latency reversal interventions [58].

We also observed an enrichment of HIV in more differentiated CD4 T cell subpopulations that have more rapid fractional replacement rates. HIV genomes were more likely to be clonally expanded in these cells as compared to their putative progenitor cells, as found by others [59–61]. Namely, effector memory cells showed the highest turnover rate and infection frequency, and were previously shown to play a major role in the waxing and waning of the HIV reservoir over time [48,62,63]. Consistent with other recent reports [45,54], we also observed identical HIV integration sites across memory subsets, further reinforcing the concept that HIV-infected cells are maintained by both clonal expansion *and* cell differentiation. Importantly, cell differentiation into effector memory phenotype was shown to help facilitate HIV latency reversal [64]. The idea that cell differentiation participates in maintaining HIV proviruses has been demonstrated elsewhere [9,65], and although we did not find identical sequences between naïve, stem-cell memory and other memory phenotypes because of sampling issues, we did find common integration sites and very strong HIV reservoir correlations in central, transitional and effector memory cells. While this may be due to HIV integration into oncogenes [22,66], only 1.3% of all integration sites in these individuals were found directly within or <10,000 base pairs distant to oncogenes.

We also found that the contribution of cellular proliferation to HIV persistence in peripheral blood is greatest among those who initiated ART later in the disease course. Those initiating ART in the first several months of infection tended to have lower fractional replacement rates of most CD4 subpopulations, which may reflect greater CD4 recovery during ART (and less of a need for homeostatic proliferation) [67]. It also may reflect less effector T cell proliferation as a consequence of decreased exposure to or better control over prevalent antigens, thus decreasing levels of bystander immune activation and inflammation [68]. Whatever the mechanistic explanation, it suggests that anti-proliferative approaches to reducing HIV reservoirs may be more effective in individuals who initiated ART late rather than early after infection.

There are several limitations of our study that deserve comment. Since all of our analyses related to HLA-DR- CD4 subpopulations, events that occur during transient activation of cells *in vivo* (such as enhanced HIV transcription) were not recorded [69,70]. All of our observations were also restricted to circulating cells. Tissue-based T cells may shed additional mechanistic light on reservoir persistence and deserve future analogous analysis. Our turnover rate estimates include both infected and uninfected cells. Thus, our implicit assumption is that HIV-infected cells have similar proliferation and fractional replacement rate characteristics as uninfected cells of the same maturational phenotype, which might not be true. Current experimental limitations preclude sorting and turnover measurements of the same exact cells, which would be necessary to interrogate this assumption. We acknowledge the fact that we have not measured intact HIV proviruses in this study as only evaluating integrated HIV DNA does not inform the replication-competency of the reservoir. One last limitation of our analysis is the lack of inclusion of female participants in our study. Since biologic sex plays an important role in modulating T cell biology [71,72], future studies focused on the impact of sex differences on HIV reservoirs and cellular turnover are warranted.

In summary, circulating HIV proviruses appear to be maintained by 3 main processes: 1) slow turnover of immature CD4 subpopulations with less HIV transcriptional activity, 2) cell differentiation from immature to mature cell types, and 3) clonal expansion of effector cells with fast replacement rates. Our study enhances the mechanistic understanding of these dynamics and highlights the challenges and opportunities for curative therapies attempting to modulate Human immunology such as shock-and-kill, block-and-lock or anti-proliferative therapies.

## Materials and methods

### Participant characteristics

Under the auspices of a study approved by the University of California San Francisco Committee on Human Research, twenty-four HIV-infected and six HIV-uninfected participants were recruited between 2015 and 2017 from the clinic-based SCOPE and OPTIONS cohorts at Zuckerberg San Francisco General Hospital. All participants were over 18 years old and provided written informed consent. The SCOPE cohort enrolls chronically HIV-infected individuals while the OPTIONS cohort enrolls individuals <12 months (before 2003) and <6 months (after 2003) after HIV antibody seroconversion. The estimated dates of infection were calculated from the first positive HIV RNA assay or antibody seroconversion tests, as previously described [73]. HIV-infected participants were required to be virally suppressed on ART upon study entry. Duration of viral suppression was estimated based on clinic records (typically assessed every 3–6 months). We stratified sampling of participants across a range of time to ART initiation (0.7 to 222 months) and duration of ART suppression (1.2 to 18.8 years), ensuring sufficient overlap in each of these parameters to allow for valid adjusted analysis of the independent contributions of ART timing and duration to biological measurements. All but one of the HIV-uninfected participants were chosen to be seropositive for CMV infection to match the CMV status of our HIV-infected cohort.

### Isolation of CD4 T cell subpopulations

All participants underwent large blood draws and leukaphereses performed as outpatients. PBMC were isolated and viably cryopreserved. Frozen PBMC were thawed and CD4 T cells enriched with the EasySep Human CD4+T Cell Negative Selection Enrichment Kit (Stemcell). CD4 T cells were stained with Live/Dead Fixable Aqua (Life Technologies) and the following monoclonal antibody cocktail: anti-CD3-FITC, anti-CD4-AlexaFluor700, anti-CCR7-PE-Cyanine7, anti-CD27-APC, anti-HLA-DR-APC H7, anti-CD57-Brilliant Violet 421, and anti-CD95-PE (Becton Dickinson) as well as anti-CD45RA-ECD (Beckman Coulter). Cells were sorted on a FACS ARIA II flow cytometer (BD Biosciences) to >97% purity, as follows: from singlets PBMC, live (Aqua-) CD3+CD4+ lymphocytes were selected based on size and granularity criteria. We then sorted either the activated (HLA-DR+) fraction, or the following subpopulations within the resting (HLA-DR-) fraction according to their expression of phenotypic markers: naïve (TN, CD45RA+CCR7+CD27+CD57-CD95-), stem-cell memory (TSCM, CD45RA+CCR7+CD27+CD57-CD95+), central memory (TCM, CD45RA−CCR7+CD27+), transitional memory (TTM, CD45RA−CCR7−CD27+), effector memory cells (TEM, CD45RA−CCR7−CD27−) and terminally-differentiated (TTD, CD45RA+CCR7-).

Dry pellets of these sorted cell populations were snap-frozen at -80°C. Flow cytometry data were analyzed on FACSDiva v8.0.1 (BD Biosciences) and FlowJo v8.7 (Tree Star).

We initially included in our study resting terminally-differentiated CD4 T cells (TTD) but given the fragility of CCR7 staining, we suspected that there may have been contamination of TN into TTD. To investigate this possibility, we measured T cell Receptor Excision Circle (TREC) content to find that TTD had an unexpectedly higher sjTREC level than TEM (see S3B Fig). Assuming that the true sjTREC content of TTD is no higher than that of TEM, we estimated a median of 20% contamination. As the sorted TTD population likely reflected a variable mix of TN and TTD cells, we could not be confident in the interpretation of data in the TTD population and have therefore excluded these data from this report.

### *In vivo* labeling with deuterated (heavy) water

Participants were provided 70% deuterated water (Sigma-Aldrich), with an oral intake of 50 ml three times per day for the first 7 days and 50 ml twice per day for the remainder of the 45 day-long labeling period. Peripheral blood was collected at baseline and at days 15, 30, and 45. Plasma was collected at all four time points and saliva samples were collected at days 7 and 21 during the first month to assess body water enrichment, which is used in the calculation of cellular replacement rate described below [17,25].

### Measurement of plasma deuterium oxide enrichment

Plasma deuterium oxide (^2^H_2_O) enrichment analysis was performed using a modified acetone equilibration [74]. Briefly, water was distilled from 100μl aliquots of plasma or saliva in inverted microvials placed in a 70°C glass bead bath. The collected distillate was reacted with 5μl acetone and 1μl 10N NaOH, and left at room temperature for 18–24 hours. Acetone was extracted from treated samples using 300ul hexane and, after drying hexane extracts with Na_2_SO_4_ crystals, acetone was analyzed by gas chromatography/mass spectrometry (GC/MS). A standard curve of percent enrichment was prepared gravimetrically from 100% enriched ^2^H_2_O. GC/MS analysis of acetone was performed by quadrupole gas chromatography/mass spectrometry (Agilent 6890/5973) using electron impact ionization mode and SIM ions of m/z 58.2 and 58.3 for acetone M0 and M+1. The column used was a DB-17MS, 30M x 0.25mm I.D., 0.25μm film thickness (J&W Scientific, Agilent).

### Measurement of deuterium enrichment in cellular genomic DNA

The stable isotope/mass spectrometric method for measuring cell proliferation has been described previously [25], and included additional precautions and controls required for working with low cell count samples. Isolated DNA (DNEasy extraction kit, Qiagen) was further hydrolyzed and dephosphorylated, and deoxyribonucleosides were derivatized using pentafluorobenzyl hydroxylamine (PFBHA) solution in acetic acid (Sigma-Aldrich). GC/MS analysis of the derivatized deoxyribose was performed as above using negative chemical ionization mode (methane) and a DB-17MS column. The SIM ions for the PFBHA derivative of deoxyribose were m/z 435, 436, and 437 respectively for M0, M1, and M2. The mole fraction of the M1 mass isotopomer was calculated as the ratio of peak areas: M1/(M0+M1+M2). Label incorporation in experimental samples was measured as the excess mole fraction of M1 (EM1) relative to the M1 mole fraction of abundance-matched unlabeled standards: [M1/(M0+M1+M2)sample]–[M1/(M0+M1+M2)standard]. The value of f, the fraction of newly synthesized DNA strands or, equivalently, the fraction of newly divided cells, was calculated as EM1/EM1*, where EM1* represents the maximal or asymptotic EM1 enrichment possible in 100% newly-divided cells under the measured mean ^2^H_2_O exposure in the participant [25]. EM1* values were calculated using measured body water enrichment and the previously determined relationship between body water enrichment and theoretical maximum possible EM1* for deoxyribose [25].

Fractional replacement rates, which can be expressed as a rate constant (*k*), were calculated by assuming a model of exponential clearance of EM1. That is, the amount remaining after time *t* can be expressed as EM1(t) = EM1(*t* = 0)exp (−*kt*). Fractional replacement rate is then estimated by fitting this equation to experimental data (using least-squares comparison with the 4 time points). From replacement rates, cellular half-lives are subsequently calculated from the newly-divided fraction (f) of cells for each subpopulation as follows: f = 1-exp (−*kt*) and *t*_1/2_ = ln(2)/*k*. These half-lives can be interpreted as the time that elapses before half of the cells of a certain subpopulation leave the pool via cell death, differentiation to other phenotypes or trafficking to other anatomic compartments. Importantly, we verified that in all cases, absolute subpopulation cell counts remained stable throughout the 45-day heavy water labeling period (S2 Fig) [25]. This ensured label clearance was not due to cell population changes, but rather turnover and dilution of deuterium labelled DNA.

### Quantification of homeostatic cytokines IL-7 and IL-15

Plasma samples were collected and immediately stored at -80°C. Thawed samples were assayed in the Enzyme-Linked ImmunoSorbent Assay Quantikine assay (R&D Systems), per the manufacturer’s recommendations. The standard curve of the IL-15 kit was further diluted to reach a range 10-times lower than indicated. All samples were run in batch and in duplicate, and read on a Molecular Devices SpectraMax M3. Reading was performed at 490nm and a 650nm correction was applied to all samples.

### Cell-associated HIV RNA quantification

Total RNA was extracted using the Allprep DNA/RNA/miRNA Universal Kit (Qiagen) with on-column DNase treatment (Qiagen RNase-Free DNase Set). HIV RNA levels were quantified with a qPCR TaqMan assay using LTR-specific primers F522-43 (5’ GCC TCA ATA AAG CTT GCC TTG A 3’; HXB2 522–543) and R626-43 (5’ GGG CGC CAC TGC TAG AGA 3’; 626–643) coupled with a FAM-BQ probe (5’ CCA GAG TCA CAC AAC AGA CGG GCA CA 3) on a StepOne Plus Real-time PCR System (Applied Biosystems, Inc.) [75]. Up to 500 ng of total RNA per sample were characterized in triplicate, and copy numbers were determined by extrapolation against a 7-point standard curve (1–10,000 copies). The input cell number in each PCR well was estimated using independent qPCR measurement of the cellular housekeeping human RPLP0 gene.

### Integrated HIV DNA quantification

Total DNA was extracted using the Allprep DNA/RNA/miRNA Universal Kit (Qiagen). Integrated HIV DNA copies were quantified using a two-step PCR reaction, as previously described [76], with modifications where isolated genomic DNA was used for PCR amplification instead of whole cell lysates. Integrated HIV DNA was pre-amplified with two Alu primers and a primer specific for the HIV LTR region, in addition to primers specific for the CD3 gene to determine cell counts. Nested qPCR was then used to amplify HIV and CD3 sequences from the first round of amplification. Specimens were assayed with up to 500 ng cellular DNA in triplicate and copy number was determined by extrapolation against a 5-point standard curve (3–30,000 copies), using extracted DNA from ACH-2 cells.

### Integration site analysis

HIV integration sites were assessed as previously described [77]. Genomic DNA (gDNA) was isolated with the Blood & Cell Culture DNA Mini Kit (Qiagen). Random fragmentation was achieved by treating 1.5 μg gDNA replicates for 20 minutes with NEBNext dsDNA fragmentase (New England Biolabs) to obtain short 200–1200 bp fragments, purified at a 1:1 ratio with AMPure XP (Agencourt Beckman Coulter). Products were end-repaired and A-tailed using the end-repair ligation module (New England Biolabs), and 10 pmol of linker was ligated to the products according to the manufacturer’s instructions. The internal HIV sequences were digested by BglII (New England Biolabs). Amplification of purified products was performed using a nested approach using described primers (Table 3), and subsequently quantitated using Nanodrop (Thermo Fisher). Up to 12 samples were pooled in equimolar amounts per library, and a sequence library was prepared using 500 ng of pooled product in the Kapa Hyper Library prep kit (Kapabiosystems). The library was further quantified using qPCR and sequenced by 300 bp paired-end sequencing on Illumina MiSeq.

**Table 3:** Linker and primer sequences used for HIV integration sites analysis.

Name	5' modification	Sequence	3' modification
Linker top	-	g*cagcgataacaatttcacggcgccactgcaggacgtac*t*g*t*t	-
Liker bot	Phosphorylated	a*cagtacgtcctgcagtggcgcgccttgactgagcttta	Dideoxycytosine
LTR 1st	Biotinylated	cttaagcctcaataaagcttgccttgag	-
Linker 1st	-	gcagcggataacaatttcacg	-
LTR 2nd	MID	tgactctggtaactagagatccctcag	-
Linker 2nd	MID	tcactgcaggacgtactgtt	-

Sequences used for primers and linkers to detect HIV integration sites. The star (*) indicates phosphorothiated nucleotide. MID stands for multiplex identifier sequence.

To identify an HIV integration site, the Miseq reads were paired and each was associated with a multiplex-identifier (MID) sequence on either end of the LTR and linker-based sequence products. Sample read pairs were subsequently checked for linker primer sequence, LTR primer sequence, and the remaining LTR nucleotide sequence ending on the nucleotide sequence CA. Matched read pairs after the LTR or linker primer sequence were trimmed to 50-base pairs and grouped according to 100% sequence match. Grouped sequences with ≥3 reads were used for chromosomal alignment using the Blat-UCSC Genome Browser (GRCH38/hg38). An HIV integration site was determined to be “clonal” if the results had ≥2 reads, and the frequency was determined by a length difference of >3 bases between the amplicons.

Integration site clonality was summarized using the Gini index (*G*): a measure of statistical dispersion originally defined to quantify unevenness in distributions of income inequality and applied to sequence clonality previously for HTLV-I virus [78]. Mathematically, it is the sum of absolute differences among all pairs of clone sizes, normalized by the average clone size ⟨*c*⟩. Thus, $G=\frac{1}{n^{2}\langle c\rangle }\sum_{i}\sum_{j}\left|c_{i}-c_{j}\right|$ where *n* is the number of clones found. Enrichment for integration of HIV in specific genes and pathways were assessed by pooling the integration sites per subpopulation using the online tool Reactome [79] as follows: the proportion of total unique HIV integration sites detected across all CD4 T cell subpopulations that fall within a given reactome (a set of linked genes, defined by an open source database of biologic pathways) and CD4 subpopulation. Specifically, the numbers in Fig 3C reflect the total number of unique integration sites within a given reactome and CD4 subpopulation divided by the total number of integration sites detected across all CD4 subpopulations, multiplied by 100.

### Measurement of sjTRECs levels

DNA was extracted using QuickExtract (Lucigen) according to manufacturer’s instructions. Briefly, cell pellets were resuspended at 10,000 cells/μL and heated at 65°C for 20 minutes with intermittent vortexing, followed by heating at 90°C for 5 minutes. DNA was then isolated at a 2:1 bead-to-cell volume ratio using Ampure XP beads (Beckman Coulter), and eluted in purified H_2_O at half the QuickExtract volume (minimum of 12 μL). sjTREC qPCR followed published methods [80]. Technical triplicates were assayed by qPCR in fast mode on a StepOnePlus (Applied Biosystems) using published human-specific sjTREC plasmids in TaqMan Fast Advanced Master Mix (Life Technologies) and quantified by comparison to sjTREC standard plasmid (kindly gifted by Drs. Gregory Sempowski and Daniel Douek) [81]. sjTREC levels were standardized to cell counts using TaqMan Copy Number Human TERT Reference Assay (ThermoFisher), according to manufacturer’s instructions. All values with undetectable wells along with distant CTs were excluded from the analysis.

### Quantification of HIV RNA transcripts

Total DNA and RNA were extracted using TRI Reagent. HIV DNA levels (R-U5-tRNA binding region) were quantified in duplicate using droplet digital PCR and normalized to copies/million cells using DNA mass (by NanoDrop). Read-through, total initiated (TAR), 5' elongated (R-U5-trRNA binding region, “Long”), polyadenylated (U3-polyA; "PolyA"), and multiply-spliced (Tat-Rev) HIV transcripts were measured in duplicate by RT-ddPCR as previously described [82], normalized to copies/μg RNA (approximately one million cells; alternative normalizations were performed using the cell counts from the sorts and DNA recovery), and divided by the HIV DNA to obtain the average level of HIV RNA/provirus.

### Statistical analysis

A two-tailed Wilcoxon matched-pairs signed rank test was used to compare cell subpopulations, and a non-parametric Mann-Whitney U-test to compare between groups. All values quoted in the text are medians and interquartile ranges. To assess associations between continuous measurements, we used non-parametric Spearman correlations as well as linear mixed models for adjusted analyses, transforming variables as appropriate to satisfy model assumptions (typically with a log_10_ transformation). Linear (or logistic) mixed models were also employed to assess differences between groups when the assumption of similar differences between groups across each subpopulation appeared valid. Analyses were performed on GraphPad Prism 7.0 (La Jolla, CA) and Stata 15.0 (College Station, TX). Hierarchical clustering was performed using the Seaborn package in Python.

## Supporting information

S1 Fig Cell sorting strategy. PBMC were sorted by flow cytometry. From singlets, lymphocytes, to live CD3+CD4+ cells, we sorted either the activated (HLA-DR+) fraction or the following subpopulations within the resting (HLA-DR-) fraction according to their expression of phenotypic markers: naïve (TN, CD45RA+CCR7+CD27+CD57-CD95-), stem-cell memory (TSCM, CD45RA+CCR7+CD27+CD57-CD95+), central memory (TCM, CD45RA−CCR7+CD27+), transitional memory (TTM, CD45RA−CCR7−CD27+), effector memory cells (TEM, CD45RA−CCR7−CD27−) and terminally-differentiated (TTD, CD45RA+CCR7-).
(TIF)

S2 Fig Characteristics of the deuterium labeling period. CD4 T cell counts, HLA-DR+ and HLA-DR- CD4 T cell subpopulations label incorporation rates were measured during the 45-day deuterium labeling study and studied at four time points: day 0 (D0), day 15 (D15), day 30 (D30) and day 45 (D45). Some data points are largely overlapping on the graphs for these participant samples. **A.** CD4 T cell counts were monitored over time and are shown as cell number per mm^3^ of peripheral blood. Longitudinal measurements from the same participants are shown with a line across the different time points, and uninfected participants are depicted in black symbols. **B.** Relative enrichment of deuterium in total HLA-DR+ CD4 T cells in six HIV-uninfected individuals over the labeling period. **C.** Fractional synthesis is expressed based on deuterium enrichment in genomic DNA and expressed at percent increase from D0 at D15, D30 and D45. It is shown for the 24 HIV-infected ART-suppressed participants in resting naïve (TN), stem-cell memory (TSCM), central memory (TCM), transitional memory (TTM) and effector memory (TEM) CD4 T cells.
(TIF)

S3 Fig Treated HIV infection does not change peripheral resting CD4 T cell proportions, recent thymic emigrants nor IL-15 levels. Peripheral frequencies and thymic output were assessed in various resting CD4 T cell populations and compared between 24 HIV-infected (HIV+) and six uninfected (HIV-) participants. We examined the homeostatic properties of the following resting CD4 T cell subpopulations: naïve (TN), stem-cell memory (TSCM), central memory (TCM), transitional memory (TTM), effector memory (TEM) and terminally-differentiated (TTD) cells. Medians and interquartile ranges [25–75%] are represented. **A.** The peripheral cell frequencies are measured and presented as percentages. Each symbol represents a participant’s sample. **B.** Central thymic output is evaluated by measuring cell-associated T cell Receptor Excision Circles (sjTRECs) in PBMC and in resting CD4 T cell subpopulations. Results are presented as copy number per million cells for each subtype, and represented on a log10 y-axis. **C.** Plasma levels of interleukin-15 (IL-15) (pg/ml) do not statistically differ between HIV+ and HIV- participants.
(TIF)

S4 Fig Resting memory CD4 T cell subpopulations’ contributions to the HIV reservoir in periphery. HIV DNA and RNA levels were measured in 24 HIV-infected ART-suppressed participants, and within the following resting CD4 T cell subpopulations: naïve (TN), stem-cell memory (TSCM), central memory (TCM), transitional memory (TTM) and effector memory (TEM) cells. **A.** The contribution of each subpopulation to the total HIV burden in resting CD4 T cells, whether integrated HIV DNA or cell-associated HIV RNA, were calculated from the infection levels and relative cell frequencies for each cell type. It reflects the contribution of each subpopulation to the proviral burden contained in all 5 resting CD4 T cell subpopulations studied here. Each subpopulation’s contribution is shown as the percentage relative to the other cell subpopulations. **B.** The relationship between integrated HIV DNA and cellular half-life is shown for TSCM cells and represented on a log10 y-axis. Integrated HIV DNA is presented as copy number per million cells, and cellular half-life is expressed in days. Each symbol represents a participant sample. Only one cell subpopulation had a significant association between integrated HIV DNA and cellular half-life, TSCM, which is presented here. **C.** Distribution and location of shared HIV integration sites among cell subpopulations.
(TIF)

S5 Fig HIV transcription initiation and elongation levels are higher in resting memory than naïve CD4 T cell subpopulations. The quantification of various sized HIV transcripts was performed in eight HIV-infected participants from whom samples were available. The analysis was carried out on the resting CD4 T cell subpopulations: naïve (TN), central memory (TCM), transitional memory (TTM) and effector memory (TEM) cells. Total cellular RNA from each subpopulation was used for a polyadenylation-RT-ddPCR assay for the TAR loop (the first region transcribed; initiated or total transcripts) and RT-ddPCR assays for HIV sequence regions suggesting transcriptional interference (“Read-through” transcripts), transcriptional initiation (“TAR”), 5’ transcriptional elongation (“Long”), completion of transcription (“PolyA”), and multiple splicing (“Tat-Rev”). Results are presented as the ratio of each cell-associated HIV RNA to total HIV DNA (both expressed as copies per million cells) to show the average level of each HIV transcript per provirus. Medians are shown on a log10 scale and only significant *p* values are indicated.
(TIF)

S6 Fig ART duration does not impact fractional replacement rate or HIV genomes. The fractional cellular replacement rate and HIV genome levels are measured in 24 HIV-infected participants, and within the following resting CD4 T cell subpopulations: naïve (TN), stem-cell memory (TSCM), central memory (TCM), transitional memory (TTM) and effector memory (TEM) cells. Antiretroviral therapy (ART) duration is estimated in years, and each symbol represents a participant’s sample. **A.** The relationship between the fractional replacement rate and ART duration was assessed in resting CD4 T cell subpopulations. The fractional replacement rate was estimated as cells produced per day. **B.** The associations between HIV genomes and ART duration are shown for all participants in the cells that make up most of the HIV reservoir, i.e. TCM, TTM, and TEM. Integrated HIV DNA and cell-associated HIV RNA are presented as copy number per million cells of a given subpopulation on a log10 y-axis. Values below the threshold of detection were calculated for each assay according to the number of cells analyzed.
(TIF)
