# Supplementary figures and images for: Relationship between CD4 T cell turnover, cellular differentiation and HIV persistence during ART
Source: PLoS Pathog. 2021 Jan 19;17(1):e1009214. doi: 10.1371/journal.ppat.1009214 (PMC7846027; doi:10.1371/journal.ppat.1009214)

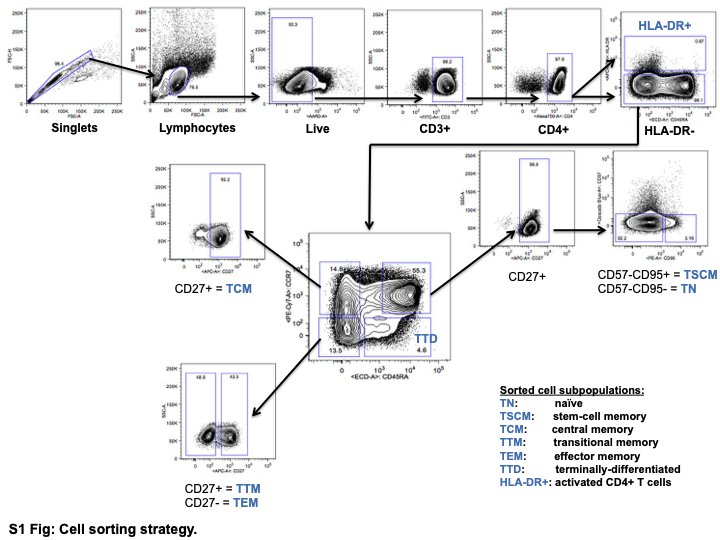

Supplement: S1 Fig — PBMC were sorted by flow cytometry. From singlets, lymphocytes, to live CD3+CD4+ cells, we sorted either the activated (HLA-DR+) fraction or the following subpopulations within the resting (HLA-DR-) fraction according to their expression of phenotypic markers: naïve (TN, CD45RA+CCR7+CD27+CD57-CD95-), stem-cell memory (TSCM, CD45RA+CCR7+CD27+CD57-CD95+), central memory (TCM, CD45RA−CCR7+CD27+), transitional memory (TTM, CD45RA−CCR7−CD27+), effector memory cells (TEM, CD45RA−CCR7−CD27−) and terminally-differentiated (TTD, CD45RA+CCR7-). (TIF) [file ppat.1009214.s001.tif]

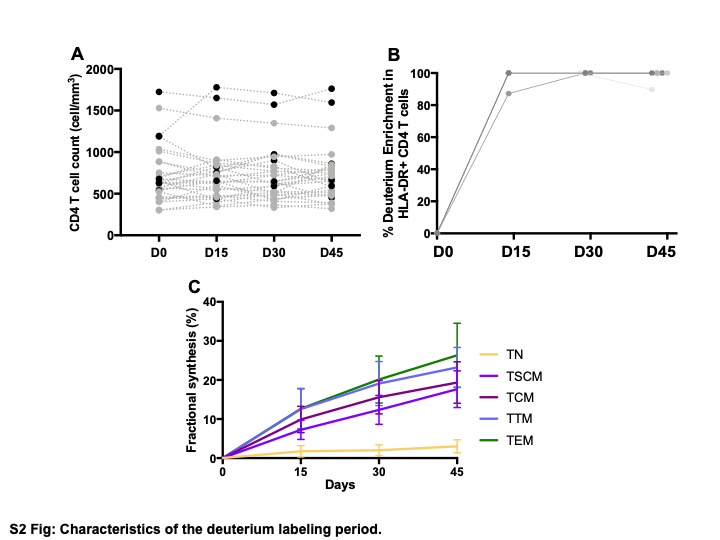

Supplement: S2 Fig — CD4 T cell counts, HLA-DR+ and HLA-DR- CD4 T cell subpopulations label incorporation rates were measured during the 45-day deuterium labeling study and studied at four time points: day 0 (D0), day 15 (D15), day 30 (D30) and day 45 (D45). Some data points are largely overlapping on the graphs for these participant samples. A. CD4 T cell counts were monitored over time and are shown as cell number per mm3 of peripheral blood. Longitudinal measurements from the same participants are shown with a line across the different time points, and uninfected participants are depicted in black symbols. B. Relative enrichment of deuterium in total HLA-DR+ CD4 T cells in six HIV-uninfected individuals over the labeling period. C. Fractional synthesis is expressed based on deuterium enrichment in genomic DNA and expressed at percent increase from D0 at D15, D30 and D45. It is shown for the 24 HIV-infected ART-suppressed participants in resting naïve (TN), stem-cell memory (TSCM), central memory (TCM), transitional memory (TTM) and effector memory (TEM) CD4 T cells. (TIF) [file ppat.1009214.s002.tif]

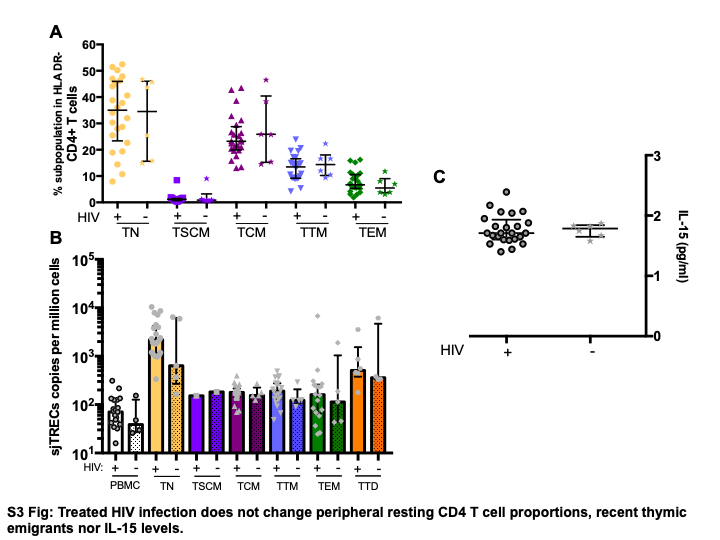

Supplement: S3 Fig — Peripheral frequencies and thymic output were assessed in various resting CD4 T cell populations and compared between 24 HIV-infected (HIV+) and six uninfected (HIV-) participants. We examined the homeostatic properties of the following resting CD4 T cell subpopulations: naïve (TN), stem-cell memory (TSCM), central memory (TCM), transitional memory (TTM), effector memory (TEM) and terminally-differentiated (TTD) cells. Medians and interquartile ranges [25–75%] are represented. A. The peripheral cell frequencies are measured and presented as percentages. Each symbol represents a participant’s sample. B. Central thymic output is evaluated by measuring cell-associated T cell Receptor Excision Circles (sjTRECs) in PBMC and in resting CD4 T cell subpopulations. Results are presented as copy number per million cells for each subtype, and represented on a log10 y-axis. C. Plasma levels of interleukin-15 (IL-15) (pg/ml) do not statistically differ between HIV+ and HIV- participants. (TIF) [file ppat.1009214.s003.tif]

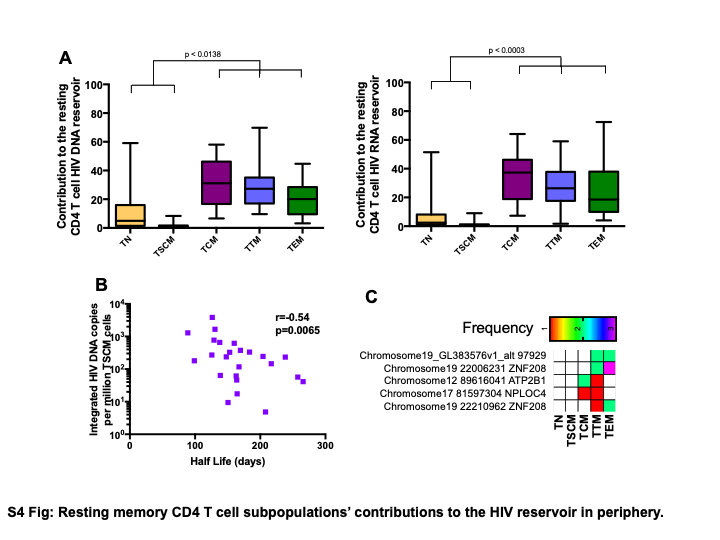

Supplement: S4 Fig — HIV DNA and RNA levels were measured in 24 HIV-infected ART-suppressed participants, and within the following resting CD4 T cell subpopulations: naïve (TN), stem-cell memory (TSCM), central memory (TCM), transitional memory (TTM) and effector memory (TEM) cells. A. The contribution of each subpopulation to the total HIV burden in resting CD4 T cells, whether integrated HIV DNA or cell-associated HIV RNA, were calculated from the infection levels and relative cell frequencies for each cell type. It reflects the contribution of each subpopulation to the proviral burden contained in all 5 resting CD4 T cell subpopulations studied here. Each subpopulation’s contribution is shown as the percentage relative to the other cell subpopulations. B. The relationship between integrated HIV DNA and cellular half-life is shown for TSCM cells and represented on a log10 y-axis. Integrated HIV DNA is presented as copy number per million cells, and cellular half-life is expressed in days. Each symbol represents a participant sample. Only one cell subpopulation had a significant association between integrated HIV DNA and cellular half-life, TSCM, which is presented here. C. Distribution and location of shared HIV integration sites among cell subpopulations. (TIF) [file ppat.1009214.s004.tif]

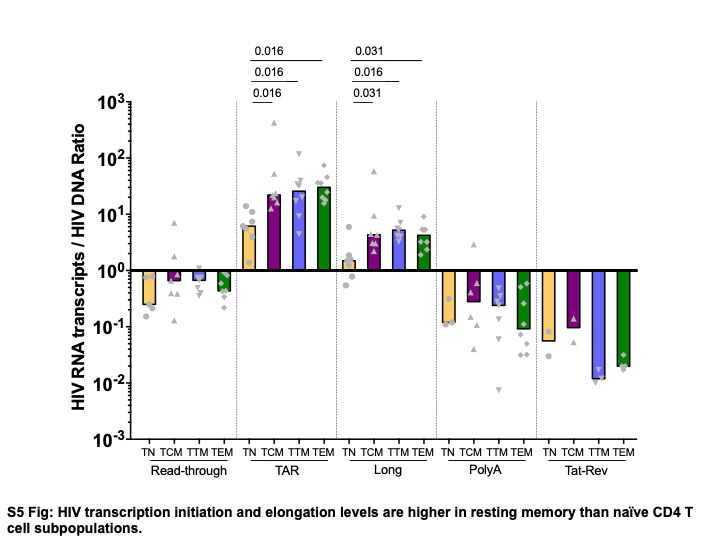

Supplement: S5 Fig — The quantification of various sized HIV transcripts was performed in eight HIV-infected participants from whom samples were available. The analysis was carried out on the resting CD4 T cell subpopulations: naïve (TN), central memory (TCM), transitional memory (TTM) and effector memory (TEM) cells. Total cellular RNA from each subpopulation was used for a polyadenylation-RT-ddPCR assay for the TAR loop (the first region transcribed; initiated or total transcripts) and RT-ddPCR assays for HIV sequence regions suggesting transcriptional interference (“Read-through” transcripts), transcriptional initiation (“TAR”), 5’ transcriptional elongation (“Long”), completion of transcription (“PolyA”), and multiple splicing (“Tat-Rev”). Results are presented as the ratio of each cell-associated HIV RNA to total HIV DNA (both expressed as copies per million cells) to show the average level of each HIV transcript per provirus. Medians are shown on a log10 scale and only significant p values are indicated. (TIF) [file ppat.1009214.s005.tif]

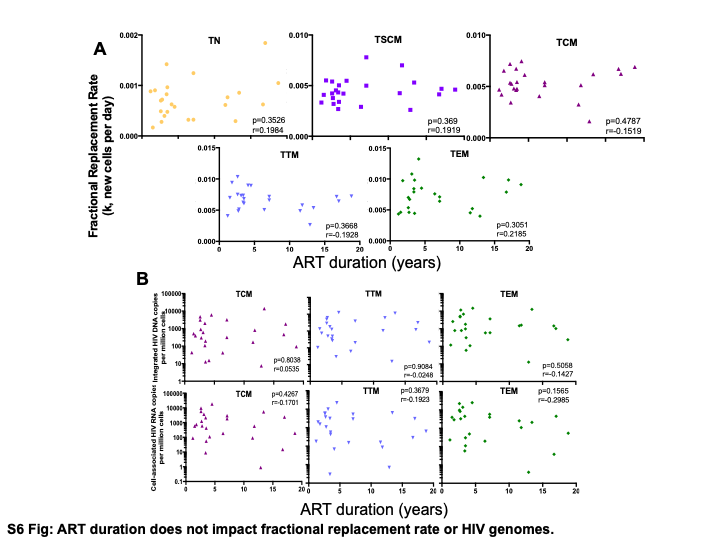

Supplement: S6 Fig — The fractional cellular replacement rate and HIV genome levels are measured in 24 HIV-infected participants, and within the following resting CD4 T cell subpopulations: naïve (TN), stem-cell memory (TSCM), central memory (TCM), transitional memory (TTM) and effector memory (TEM) cells. Antiretroviral therapy (ART) duration is estimated in years, and each symbol represents a participant’s sample. A. The relationship between the fractional replacement rate and ART duration was assessed in resting CD4 T cell subpopulations. The fractional replacement rate was estimated as cells produced per day. B. The associations between HIV genomes and ART duration are shown for all participants in the cells that make up most of the HIV reservoir, i.e. TCM, TTM, and TEM. Integrated HIV DNA and cell-associated HIV RNA are presented as copy number per million cells of a given subpopulation on a log10 y-axis. Values below the threshold of detection were calculated for each assay according to the number of cells analyzed. (TIF) [file ppat.1009214.s006.tif]
